# Moderated by personal perception: The preventive relationship between home HIIT dance and depression during the COVID-19 pandemic in China

**DOI:** 10.3389/fpubh.2023.1117186

**Published:** 2023-02-09

**Authors:** Yifan Hu, KwanJung Son, Zheng Yang, Yufei Mao

**Affiliations:** ^1^Department of Dance, College of Performing Arts and Sport, Hanyang University, Seoul, Republic of Korea; ^2^Department of Education, Changshu Institute of Technology, Suzhou, China; ^3^School of Communication, Soochow University, Suzhou, China

**Keywords:** HIIT dance, depression, COVID-19 lockdown, health belief model, mediation effect

## Abstract

**Introduction:**

Lockdowns during the COVID-19 pandemic were believed to greatly increase the risk of depression among isolated residents in both China and in Western countries. How to effectively reduce this risk has become one of the key issues in the field of public mental health.

**Methods:**

The present study seeks to examine the preventive relationship between doing home HIIT dance—which became popular during Shanghai's COVID-19 lockdown in 2022—and depression, and how such a preventive relationship has been mediated by different personal perception factors using an online survey with 528 samples.

**Results:**

The preventive relationship between doing home HIIT dance and depression was differently mediated by residents' personal perception factors, such as perceived benefits, severity, and self-efficacy, based on the health belief model.

**Discussion:**

These results deepen the research on the psychological effects of doing home HIIT dance on preventing depression, especially in the COVID-19 lockdown period, emphasizing the possible moderation effects of different self-perception factors.

## 1. Introduction

During the COVID-19 pandemic, China implemented city-lockdown management in many cities, such as Wuhan, Shanghai, and Chengdu until September 2022. At time of writing, the city lockdown policy is still being implemented in some areas, and some Chinese residents have to be quarantined at home for a long time. Many studies have found that long-term isolation or quarantine in a fixed area (such as at home) can significantly increase the risk of depression among residents (1–4). During the lockdowns, the lack of necessary and timely psychological intervention and treatment also increased the severity of depression cases (5, 6).

Many studies have found that moderate fitness and exercise can reduce the risk of depression (7–10). Lack of physical activity, including exercise, has been found to be associated with depression and increased anxiety levels during COVID-19 (11). In the Shanghai lockdown in the first half of 2022, a group of Chinese entertainers, represented by dancer and actor Liu Genghong, began to livestream HIIT dance at home on many Chinese social media platforms, such as Douyin, which led to many residents in lockdown areas following the exercises at home. After that, a large number of Chinese entertainment stars also began to teach HIIT dance on various Chinese social media platforms, instigating a popular trend. Many studies have also found the positive physiological and psychological effects of doing HIIT dance (12, 13). But does this kind of home HIIT exercise reduce the risk of depression, which was amplified during the COVID-19 lockdown? And if so, do other perceptual factors play a role? To explore these questions, 528 residents quarantined in the Shanghai lockdown during the first half of 2022 were surveyed, and responses combined with the health belief model to understand the data. The results show that doing home HIIT dance does have preventive effects on depression experienced during China's COVID-19 lockdown and that it was differently mediated by residents' personal perception factors, such as perceived benefits, severity, and self-efficacy. Perceived benefits and perceived severity can effectively magnify the preventive effect of doing home HIIT on depression, while low perceived self-efficacy can prevent this preventive effect.

## 2. COVID-19 lockdown in China and the magnified risk of depression

On 23 January 2020, the Chinese government agreed on a lockdown policy in “The Silent City” of Wuhan, where China's first COVID-19 cases were discovered. This lockdown lasted 76 days, which included many strict requirements: all the city's public transport, including subway, ferry and long-distance passenger transport was suspended and citizens were not allowed to leave Wuhan without special reasons. All places of public entertainment, including cinemas, restaurants, amusement parks, and shopping malls, were closed. Residents were told not to leave their homes and all living materials were arranged by the government. The lockdown was believed to have effectively prevented the spread of COVID-19 in Wuhan and beyond. This experience was learned from when the virus spread to many other cities in China. On 28 March 2022, due to the sudden large-scale prevalence of COVID-19, Shanghai began to implement a lockdown policy. During the Shanghai lockdown, residents were also required to stay at home. All living materials were also purchased uniformly and transported by special personnel arranged by the government. This lockdown lasted for 75 days. China is currently implementing a “Dynamic Reset” policy (also called dynamic zero-COVID-19 policy) and lockdown policies for those cities with COVID-19 outbreaks.

The lockdowns during the pandemic were found to greatly increase the risk of depression and other psychological diseases among residents in the locked down cities (14–16). This conclusion has been reflected in multinational data. For instance, Khubchandani et al. (17) found an increased rate of depression and anxiety after the COVID-19 lockdowns in the USA; Schwinger et al. (18) also found increases in anxiety and depressive symptom and declines in autonomy, wellbeing and relatedness satisfaction in Germany because of the lockdowns. Wang et al. (16) and Du et al. (19) found a significant increase in the rate of depression in Chinese adolescents and adults, respectively. For Chinese students, COVID-19 on-campus quarantine increased diagnoses of depression from 9.1 to 36.1% (20). The rapid growth of psychological diseases such as depression caused by the COVID-19 lockdown or quarantine has become a common problem globally.

## 3. Preventive effect of exercise on depression and its perceptual factors

Many studies have already pointed out that sport or exercise can protect against symptoms of mental disorders, including depression, especially for adolescents and youths (21–24). Among many kinds of exercise, as a combination of physiological movement and dance music aesthetics (exercise and entertainment), high-intensity interval training (HIIT) dance has been demonstrated to have physiological and psychological benefits (12, 13), which may play a better role in the prevention of mental diseases. Some studies have demonstrated the preventive effects of HIIT dance on depression and other mental disorders, and further suggest that HIIT dance may be an easy and effective exercise mode for people with mental disorders (12, 25). But the literature evaluating the effects of HIIT on people with mental disorders is still in its infancy (26). As mentioned above, during lockdowns, when it was difficult to achieve regular outdoor fitness and other exercise, home HIIT dance became one of the most common forms of exercise for quarantined residents. But can this form of home fitness effectively cope with or prevent the increased risk of depression caused by the lockdowns? This question has not been answered effectively, especially in the Chinese context, where COVID-19 lockdowns are still in progress. This is also the main research question of this study.

**Figure 1:**
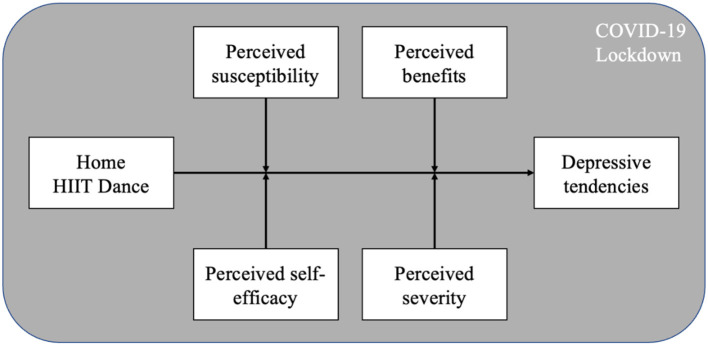
Model of the relationship between home HIIT dance and depression during COVID-19 lockdown.

According to the health belief model, people's health-related behaviors are highly affected by their perception factors, such as perceived susceptibility, severity, benefits and self-efficacy (27–29). Specifically, the preventive effect of exercise on depression and other mental disorders has also been found to be moderated by individual cognitive factors, such as personal risk perception and benefit perception (30–32). Other behavior research theories, such as the Theory of Planned Behavior (TPB), which is also widely used in health-related behaviors research, also indicate that the health behaviors and effects of individuals are affected by personal perception factors, including the health behaviors adopted to prevent and treat depression (33, 34). People with different perceptions on exercise and mental disorders will have different responses to the effects of exercise on depression. For instance, Warner et al. (35) found that Canadians with higher depression risk perception are more likely to benefit from self-help behaviors, including doing exercise. Bodin and Martinsen (36) also found that people with higher perceptions of self-efficacy tended to have better control in relation to exercise, which could further magnify the preventive effect of exercise on depression. For HIIT dance, it has also been found that personal perceptions such as perceived susceptivity, severity, benefits, and self-efficacy also significantly moderate its effects to prevent mental disorders (37, 38). Although the moderated effect of personal perceptions in the preventive effect of exercise on depression and other mental disorders has been widely found, its specific application during the COVID-19 lockdown has not been fully studied, especially in the Chinese context. Therefore, based on the above, we proposed the following two main research questions for this study (Figure 1):

RQ1. During the Chinese COVID-19 lockdown, was there a significant preventive relationship between doing home HIIT and depression among quarantined Chinese residents?

RQ2. If so, is the preventive relationship of doing home HIIT on the depression of quarantined Chinese residents moderated by their personal perception factors, including perceived susceptibility, severity, benefits, and self-efficacy?

## 4. Method and sample

A fixed-point survey was conducted *via* a Chinese professional survey company, Wenjuanxing, when Shanghai was locked down from 28 March to 12 June 2022. The study followed the ethical guidelines of the authors' universities. A total of 528 Shanghai residents who experienced this lockdown were surveyed and all participation was voluntary and paid. All participants were comprehensively informed about the purpose of the study and they all provided informed consent electronically. This study did not contain any personally identifying details.

The survey involves four variables: (1) social-demographic characteristics, including age, gender, educational level, income level; (2) frequency of home HIIT-dance participation during lockdown; (3) depression tendency assessment using the Zung Self-Rating Depression Scale (SDS) (39); and (4) individual cognition, including perceived susceptivity and severity of depression during lockdown, perceived benefits of the preventive relationship between HIIT-dance and depression, and perceived self-efficacy of doing HIIT-dance; this part and its measure were mainly based on the health belief model (27) (Table 1). The individual cognition factors were measured based on the potential relationship between doing HIIT-dance and depression tendency. For instance, “perceived susceptivity” measures the residents' perception of the possibility of suffering from depression during the lockdown period; “perceived severity” measures the residents' perception of the severity of suffering depression during lockdown; “perceived benefits” measures residents' perception of the effect of doing HIIT-dance on reducing the risk of depression during lockdown; and “perceived self-efficacy” measures the residents' perception of the feasibility of their psychological and objective conditions for doing HIIT-dance during lockdown.

**Table 1:** Basic characteristics of respondents.

Age	Under 18	3 (0.6%)
	18–30	212 (40.2%)
	30–50	285 (54%)
	51 and above	28 (5.3%)
Gender	Male	255 (48.3%)
	Female	273 (51.7%)
Educational level	Junior high school and below	7 (1.3%)
	High school/technical secondary school	35 (6.6%)
	Undergraduate	424 (80.3%)
	Postgraduate and above	62 (11.7%)
Income level	3,000 and lower	25 (4.7%)
	3,000–5,000	33 (6.3%)
	5,000–8,000	99 (18.8%)
	8,000–10,000	132 (25.0%)
	10,000–15,000	149 (28.2%)
	15,000-20,000	53 (10.0%)
	***M***	***SD***
Depression tendency	2.31	0.597
Frequency of home HIIT-dance participation	3.17	1.217
Perceived susceptivity	2.74	1.079
Perceived severity	2.45	0.675
Perceived benefits	4.09	0.715
Perceived self-efficacy	4.14	0.686

Multiple linear regression (MLR) and a simple slope test (SST) were used to analyse the data using SPSS 26.0. MLR was used to identify the factors associated with depression tendency, while SST was used to identify the moderation effects of individual cognition factors on the possible preventive relationship between doing HIIT-dance and depression.

## 5. Results

### 5.1. Significantly preventive relationship between doing home HIIT dance and depression of quarantined Chinese residents during lockdown

Firstly, as displayed in Table 2, some demographic factors are significantly relevant to the Chinese residents' depression index, such as age and income level. This study finds that during the COVID-19 lockdown period in Shanghai, older people and those with higher income were less likely to suffer from depression (significant negative correlations). According to the data, the depression tendency of Chinese residents during the lockdown is significantly related to their worries about income (40). The income level of young people is more unstable than that of the elderly, and their savings are also lower; people with lower income levels are more likely to worry about the impact of lockdown on their income, thus showing a clearer tendency toward depression. The regression analysis results also show that the frequency of Chinese residents' home HIIT-dance during COVID-19 lockdown is significantly inversely proportional to their depression index (*t* = −3.578, *p* < 0.001). This indicates that the more often the Chinese residents engage in home HIIT-dance, the less likely they are to suffer from depression during lockdown. There is a preventive association between doing home HIIT-dance and suffering from depression. This finding is consistent with the results of other studies on the relationship between home fitness and mental or psychological disorders during COVID-19 lockdown and beyond (22, 25, 26, 41), and further expands the effective home-fitness exercises to include HIIT-dance.

**Table 2:** Hierarchical regression results of associated factors with depression.

	**Step 1**	**Step 2**
Age	−3.657^***^	−4.067^***^
Gender	−1.108	−0.213
Educational Level	1.019	1.296^*^
Income Level	−3.580^***^	−2.680^***^
Frequency of home HIIT-dance		−3.578^***^
*R^2^*	0.241	0.282
Adjusted *R^2^*	0.374	0.357

Dependent Variable: Depression Index.

p < 0.10;

* p < 0.05;

^**^p < 0.01;

*** p < 0.001.

### 5.2. Different perception factors differentially moderate the preventive relationship between home HIIT-dance and depression of quarantined Chinese residents during the lockdown

**Figure 2:**
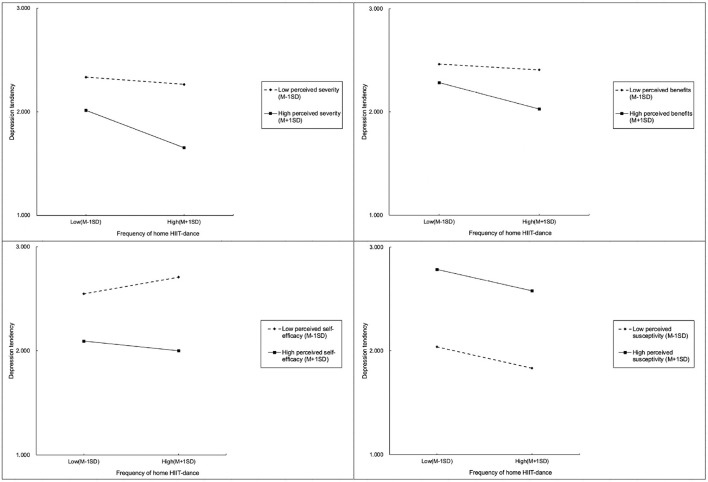
Simple slope test results of the mediated effects of personal perceived factors.

As shown in Figure 2, the simple slope test results show that the preventive relationship between doing home HIIT and depression of quarantined Chinese residents during the COVID-19 lockdown is indeed differently moderated by personal perception factors. Higher perceived severity and perceived benefits can effectively amplify the preventive association of doing home HIIT and depression during lockdown, which means if those Chinese residents can more clearly perceive that depression during isolation will bring serious consequences, or they can more clearly perceive that engaging in home HIIT will bring psychological benefits, the preventive association between doing home HIIT and suffering from depression will be more significant (upper-left and upper-right of Figure 2).

Perceived self-efficacy plays a more complex moderation role on the preventive association between doing home HIIT and the depression of quarantined Chinese residents. If the residents have a clear sense that they can effectively engage in home HIIT in their living environment it can significantly prevent their experiences of depression. Conversely, if they feel that doing home HIIT is impossible or difficult, then doing home HIIT will not prevent depression and it may also increase the risk of depression. This moderation effect of perceived self-efficacy may be relevant to the psychological principle that engaging in activities with low feelings of self-efficacy will increase the psychological burden of subjects (42, 43) (lower-left of Figure 2).

Lastly, according to the simple slope test results, perceived susceptivity does not play a clear moderation role on the preventive association between doing home HIIT and the depression of quarantined Chinese residents (lower-right of Figure 2). In conclusion, the preventive association between doing home HIIT and the depression of quarantined Chinese residents is indeed differently moderated by personal perception factors, including perceived susceptibility, severity, benefits and self-efficacy.

## 6. Discussion

The present study sought to examine the psychological association between doing home HIIT dance and the depression of Chinese residents during COVID-19 lockdowns. Based on the statistical results and the theory of health belief model, we found that there is indeed a preventive association between doing home HIIT dance and depression among Chinese residents during COVID-19 lockdowns—the Chinese residents who more frequently practiced home HIIT dance were less likely to suffer from depression. The results overall are in line with our suppositions, indicating that the preventive association between doing home HIIT and depression among quarantined Chinese residents is indeed differently moderated by personal perception factors. However, although most of the correlations and verifications were statistically significant, there may be significant differences in the causes of the effects of those mediator variables.

For the two mediator variables of perceived severity and perceived benefits, it is easy to understand their moderation mechanisms. Many studies have pointed out that, in this risk society, risk-related perception significantly regulates people's psychological state and their behavior (44, 45). According to risk perception theory, perceived severity and perceived benefits are two important dimensions of people's risk perception (46–48). When we can clearly perceive that the negative consequences of a thing or behavior may be very serious, we are more inclined to have a response that can avoid it; and when we can clearly perceive the significant benefits of a thing or behavior, we are more inclined to adopt it (49). This is the basic mechanism of perceived severity and perceived benefits in people's health behavior tendency, according to the health belief model (50). Therefore, when Chinese residents can more clearly perceive the severity of the consequences of their depression during lockdown, they may be more inclined to seek relevant coping measures to avoid depression and be more active in the implementation of such measures to improve their effect, such as by doing home HIIT dance. Correspondingly, when they can more clearly perceive the effects of avoiding depression or other relevant benefits of certain measures, they may also be more active in doing them, thus further enlarging their positive effect. Consequently, to reduce depression through doing home HIIT dance during lockdowns and to further magnify its effect, we can add the relevant information about the severity of the consequences of depression in this special period and the effectiveness of doing home HIIT dance in avoiding depression, in publicizing this activity and its benefits.

A somewhat unexpected outcome is the negative mediation effect of perceived self-efficiency on the preventive association between doing home HIIT and depression of quarantined Chinese residents. Perceived self-efficacy refers to the psychological state in which individuals perceive that they can complete a task (51). When perceived self-efficiency is low, carrying out that task will bring a greater psychological burden, and further influence the completion and effect of that task (52). In this study, when Chinese residents have low perceived self-efficiency for home HIIT dance—believing that the environment and physical conditions are unfavorable for such exercise—forcing them to do home HIIT dance exercise may increase their psychological burden and further weaken its preventive association with depression. Many studies have found that during COVID-19, residents' perceived self-efficiency in many fields saw a significant decline, including online learning, food choice, and exercise (53–55). Therefore, to avoid the negative effect of low perceived self-efficiency on the preventive association between doing home HIIT and depression, more effective publicity and demonstrations are needed to improve the perceived self-efficiency of residents and to encourage them to engage in such exercise.

This study has deepened research on the psychological associations between doing home HIIT dance and preventing depression, especially in the COVID-19 lockdown period, emphasizing the possible moderation effects of different self-perception factors. The health belief model used in this study only provides one of many perspectives but this study has provided a new line of thought: it is already well-known that doing exercise, including home HIIT dance, can reduce the risk of depression, but it is less clear what perceptual factors have an effect on this outcome. Therefore, if we want to further understand such effects, we can start from a more detailed level of personal perception in future research and discussion.

## 7. Conclusion

This study observes that doing home HIIT dance has a preventative association with depression, especially during the COVID-19 lockdown period in China. But such an association is differently mediated by different personal perception factors, such as perceived severity, benefits and self-efficiency. Regarding the most important limitation of this study, we acknowledge that, due to the quasi-longitudinal questionnaire method, participants' estimations about their participation and impression of home HIIT dance during Shanghai's lockdown may be biased by memory and contrast effects as participants must recall their perceptions before or during home HIIT dance. Moreover, the samples are all from Shanghai, focusing on their psychological statuses and home exercise, which is not representative of the wider Chinese population, limiting the generalizability of the findings of this study. As a pilot study, our data still provides insights into the relations between personal perceptions and how to reduce the psychological consequences of the COVID-19 lockdown in China.

## Data availability statement

The raw data supporting the conclusions of this article will be made available by the authors, without undue reservation.

## Ethics statement

The studies involving human participants were reviewed and approved by Hanyang University; Soochow University. The patients/participants provided their written informed consent to participate in this study.

## Author contributions

ZY and YH: conceptualization and data analysis. ZY and YM: methodology. ZY: writing. KS: review and editing. All authors contributed to the article and approved the submitted version.

## References

[1] Benke C Autenrieth LK Asselmann E Pané-Farré CA. Lockdown, quarantine measures, and social distancing: Associations with depression, anxiety and distress at the beginning of the COVID-19 pandemic among adults from Germany. Psychiatry Res. (2020) 293:113462. 10.1016/j.psychres.2020.113462 32987222 PMC7500345

[2] Xin M Luo S She R Yu Y Li L Wang S. Negative cognitive and psychological correlates of mandatory quarantine during the initial COVID-19 outbreak in China. Am Psychologist. (2020) 75:607. 10.1037/amp0000692 32673008

[3] Samrah SM Al-Mistarehi AH Aleshawi AJ Khasawneh AG Momany SM Momany BS. Depression and coping among COVID-19-infected individuals after 10 days of mandatory in-hospital quarantine, Irbid, Jordan. Psychol Res Behav Manag. (2020) 13:823. 10.2147/PRBM.S267459 33116970 PMC7547909

[4] Konstantopoulou G Raikou N. Clinical evaluation of depression in university students during quarantine due to COVID-19 pandemic. Eur J Public Health Stud. (2020) 3:1–13. 10.46827/ejphs.v3i1.65

[5] Cavicchioli M Ferrucci R Guidetti M Canevini MP Pravettoni G Galli F. What will be the impact of the Covid-19 quarantine on psychological distress? Considerations based on a systematic review of pandemic outbreaks. Healthcare. (2021) 9:101. 10.3390/healthcare9010101 33477981 PMC7835976

[6] Hamaideh SH Al-Modallal H Tanash MA Hamdan-Mansour3 A. Depression, anxiety and stress among undergraduate students during COVID-19 outbreak and “home-quarantine”. Nursing Open. (2022) 9:1423–31. 10.1002/nop2.918 33988913 PMC8242644

[7] Strawbridge WJ Deleger S Roberts RE Kaplan GA. Physical activity reduces the risk of subsequent depression for older adults. Am J Epidemiol. (2002) 156:328–34. 10.1093/aje/kwf047 12181102

[8] Donaghy ME. Exercise can seriously improve your mental health: Fact or Fiction? Adv Physiother. (2007) 9:76–88. 10.1080/14038190701395838

[9] Teychenne M Ball K Salmon J. Physical activity and likelihood of depression in adults: A review. Prev Med. (2008) 46:397–411. 10.1016/j.ypmed.2008.01.009 18289655

[10] Pascoe MC Parker AG. Physical activity and exercise as a universal depression prevention in young people: A narrative review. Early Interv Psychiatry. (2019) 13:733–9. 10.1111/eip.12737 30302925

[11] Puccinelli PJ da Costa TS Seffrin A de Lira CAB Vancini RL Nikolaidis PT. Reduced level of physical activity during COVID-19 pandemic is associated with depression and anxiety levels: An internet-based survey. BMC Public Health. (2021) 21:1–11. 10.1186/s12889-021-10470-z 33648487 PMC7919983

[12] Korman N Armour M Chapman J Rosenbaum S Kisely S Suetani S. High intensity interval training (HIIT) for people with severe mental illness: A systematic review and meta-analysis of intervention studies–considering diverse approaches for mental and physical recovery. Psychiatry Res. (2020) 284:112601. 10.1016/j.psychres.2019.112601 31883740

[13] Martland R Korman N Firth J Vancampfort D Thompson T Stubbs B. Can high-intensity interval training improve mental health outcomes in the general population and those with physical illnesses? A systematic review and meta-analysis. Br J Sports Med. (2022) 56:279–91. 10.1136/bjsports-2021-103984 34531186

[14] Le HT Lai AJX Sun J Hoang MT Vu LG Pham HQ. Anxiety and depression among people under the nationwide partial lockdown in Vietnam. Front Public Health. (2020) 8:589359. 10.3389/fpubh.2020.589359 33194995 PMC7658379

[15] Rehman U Shahnawaz MG Khan NH Kharshiing KD Khursheed M Gupta K. Depression, anxiety and stress among Indians in times of COVID-19 lockdown. Community Ment Health J. (2021) 57:42–8. 10.1007/s10597-020-00664-x 32577997 PMC7309680

[16] Wang D Zhao J Ross B Ma Z Zhang J Fan F. Longitudinal trajectories of depression and anxiety among adolescents during COVID-19 lockdown in China. J Affect Disord. (2022) 299:628–35. 10.1016/j.jad.2021.12.086 34952127 PMC8691948

[17] Khubchandani J Sharma S Webb FJ Wiblishauser MJ Bowman SL. Post-lockdown depression and anxiety in the USA during the COVID-19 pandemic. J Public Health. (2021) 43:246–53. 10.1093/pubmed/fdaa250 33426559 PMC7928742

[18] Schwinger M Trautner M Kärchner H Otterpohl N. Psychological impact of corona lockdown in Germany: Changes in need satisfaction, well-being, anxiety, and depression. Int J Environ Res Public Health. (2020) 17:9083. 10.3390/ijerph17239083 33291377 PMC7731307

[19] Du J Mayer G Hummel S Oetjen N Gronewold N Zafar A. Mental health burden in different professions during the final stage of the COVID-19 lockdown in China: Cross-sectional survey study. J Med Internet Res. (2020) 22:e24240. 10.2196/24240 33197231 PMC7713530

[20] Kuang Y Ma D Lan Z Zeng S Li Y Shang M. The Rapid Change of Mental Health in College Students After On-campus Quarantine in Shanghai 2022 COVID Lockdown, 12 July 2022, PREPRINT (Version 1). Shanghai: Research Square. (2022). 10.21203/rs.3.rs-1848919/v1 PMC10196357 37213647

[21] Eime RM Young JA Harvey JT Charity MJ Payne WR. A systematic review of the psychological and social benefits of participation in sport for children and adolescents: informing development of a conceptual model of health through sport. Int J Behav Nutr Phys Act. (2013) 10:1–21. 10.1186/1479-5868-10-98 23945179 PMC3751802

[22] Panza MJ Graupensperger S Agans JP Doré I Vella SA Evans MB. Adolescent sport participation and symptoms of anxiety and depression: A systematic review and meta-analysis. J Sport Exer Psychol. (2020) 42:201–18. 10.1123/jsep.2019-0235 32438339 PMC7679280

[23] Biddle SJ Ciaccioni S Thomas G Vergeer I. Physical activity and mental health in children and adolescents: An updated review of reviews and an analysis of causality. Psychol Sport Exerc. (2019) 42:146–55. 10.1016/j.psychsport.2018.08.011

[24] Liddle SK Deane FP Batterham M Vella SA. A brief sports-based mental health literacy program for male adolescents: A cluster-randomized controlled trial. J Appl Sport Psychol. (2021) 33:20–44. 10.1080/10413200.2019.1653404

[25] Borrega-Mouquinho Y Sánchez-Gómez J Fuentes-García JP Collado-Mateo D Villafaina S. Effects of high-intensity interval training and moderate-intensity training on stress, depression, anxiety, and resilience in healthy adults during coronavirus disease 2019 confinement: A randomized controlled trial. Front Psychol. (2021) 12:643069. 10.3389/fpsyg.2021.643069 33716913 PMC7943442

[26] Ribeiro JA Schuch FB Vargas KFM Müller PT Boullosa D. A rapid review of randomized trials assessing the effects of high-intensity interval training on depressive symptoms in people with mental illness. Int J Environ Res Public Health. (2022) 19:10581. 10.3390/ijerph191710581 36078299 PMC9518083

[27] Champion VL Skinner CS. The health belief model. Health Behav Health Educ. (2008) 4:45–65. 10.1111/j.1746-1561.1983.tb04047.x 6550149

[28] Castonguay J Filer CR Pitts MJ. Seeking help for depression: Applying the health belief model to illness narratives. Southern Commun J. (2016) 81:289–303. 10.1080/1041794X.2016.1165729

[29] Bressington DT Cheung TCC Lam SC Suen LKP Fong TKH Ho HSW. Association between depression, health beliefs, and face mask use during the COVID-19 pandemic. Front Psychiatry. (2020) 11:571179. 10.3389/fpsyt.2020.571179 33192697 PMC7642487

[30] Prins MA Verhaak PF Bensing JM van der Meer K. Health beliefs and perceived need for mental health care of anxiety and depression—The patients' perspective explored. Clin Psychol Rev. (2008) 28:1038–58. 10.1016/j.cpr.2008.02.009 18420323

[31] Bartholomew JB Ciccolo JT Spirduso W Poon L Chodzo-Zajko W. Exercise, depression, and cognition. Exerc Mediat Effects Cogn. (2008) 2:50–80. 10.5040/9781492597315.ch-003

[32] Mathersul DC Rosenbaum S. The roles of exercise and yoga in ameliorating depression as a risk factor for cognitive decline. Evid Bas Complement Alternat Med. (2016) 2016:1–9. 10.1155/2016/4612953 28044084 PMC5156813

[33] Bohon LM Cotter KA Kravitz RL Cello Jr PC Fernandez y Garcia E. The theory of planned behavior as it predicts potential intention to seek mental health services for depression among college students. J Am Coll Health. (2016) 64:593–603. 10.1080/07448481.2016.1207646 27386898 PMC5181847

[34] Zorrilla MM Modeste N Gleason PC Sealy DA Banta JE Trieu SL. Depression and help-seeking intention among young adults: The theory of planned behavior. Am J Health Educ. (2019) 50:236–44. 10.1080/19325037.2019.161601432044756

[35] Warner E Nannarone M Smail-Crevier R Manuel D Lashewicz B Patten S. The relationship between depression risk perception and self-help behaviours in high risk Canadians: A cross-sectional study. BMC Public Health. (2020) 20:1–12. 10.1186/s12889-020-08983-0 32505198 PMC7276077

[36] Bodin T Martinsen EW. Mood and self-efficacy during acute exercise in clinical depression. A randomized, controlled study. J Sport Exerc Psychol. (2004) 26:623–33. 10.1123/jsep.26.4.62311142075

[37] Paolucci EM Loukov D Bowdish DM Heisz JJ. Exercise reduces depression and inflammation but intensity matters. Biol Psychol. (2018) 133:79–84. 10.1016/j.biopsycho.2018.01.015 29408464

[38] Yu H He J Szumilewicz A. Pregnancy activity levels and impediments in the era of COVID-19 based on the health belief model: A cross-sectional study. Int J Environ Res Public Health. (2022) 19:3283. 10.3390/ijerph19063283 35328974 PMC8954454

[39] Zung WW. Zung self-rating depression scale and depression status inventory. In: Assessment of Depression. Berlin; Heidelberg: Springer (1986). p. 221–31. 10.1007/978-3-642-70486-4_21

[40] Kar SK Oyetunji TP Prakash AJ Ogunmola OA Tripathy S Lawal MM. Mental health research in the lower-middle-income countries of Africa and Asia during the COVID-19 pandemic: A scoping review. Neurol Psychiatry Brain Res. (2020) 38:54–64. 10.1016/j.npbr.2020.10.003 33162686 PMC7598562

[41] Amorosi M. Correlation between sport and depression. Psychiatria Danubina. (2014) 26:208–10. 10.1177/104747570201700418 25413542

[42] McAuley E Courneya KS Lettunich J. Effects of acute and long-term exercise on self-efficacy responses in sedentary, middle-aged males and females. Gerontologist. (1991) 31:534–42. 10.1093/geront/31.4.534 1894158

[43] Caldwell K Harrison M Adams M Quin RH Greeson J. Developing mindfulness in college students through movement-based courses: Effects on self-regulatory self-efficacy, mood, stress, and sleep quality. J Am Coll Health. (2010) 58:433–42. 10.1080/07448480903540481 20304755 PMC2879280

[44] Janmaimool P Watanabe T. Evaluating determinants of environmental risk perception for risk management in contaminated sites. Int J Environ Res Public Health. (2014) 11:6291–313. 10.3390/ijerph110606291 24937530 PMC4078580

[45] Fabiansson C Fabiansson S. Food and the Risk Society: The Power of Risk Perception. London: Routledge. (2016). 10.4324/9781315582627

[46] Oltedal S Moen BE Klempe H Rundmo T. Explaining risk perception: An evaluation of cultural theory. Rotunde. (2004) 85:1–33. 10.1080/135753097348447

[47] Barnett DJ Balicer RD Blodgett DW Everly Jr GS Omer SB Parker CL. Applying risk perception theory to public health workforce preparedness training. J Public Health Manag Practice. (2005) 11:S33–7. 10.1097/00124784-200511001-00006 16205540

[48] Korstanje M. Re-visiting risk perception theory in the context of travel. E-review Tour Res. (2009) 7:1–2. 10.1111/j.1708-8305.2012.00663.x 23279223

[49] Joseph G Burke NJ Tuason N Barker JC Pasick RJ. Perceived susceptibility to illness and perceived benefits of preventive care: An exploration of behavioral theory constructs in a transcultural context. Health Educ Behav. (2009) 36(5_suppl):71S−90S. 10.1177/1090198109338915 19805792 PMC2941192

[50] Paek HJ Hove T. Risk perceptions and risk characteristics. In:Nussbaum J, editor. Oxford Research Encyclopedia of Communication. Oxford: Oxford University Press. (2017) 23–8. 10.1093/acrefore/9780190228613.013.283

[51] Bandura A. Perceived self-efficacy in cognitive development and functioning. Educ Psychol. (1993) 28:117–48. 10.1207/s15326985ep2802_3

[52] Leganger A Kraft P R?ysamb E. Perceived self-efficacy in health behaviour research: Conceptualisation, measurement and correlates. Psychol Health. (2000) 15:51–69. 10.1080/08870440008400288

[53] Leyton-Román M de la Vega R Jiménez-Castuera R. Motivation and commitment to sports practice during the lockdown caused by COVID-19. Front Psychol. (2021) 2020:3846. 10.3389/fpsyg.2020.622595 33505343 PMC7830375

[54] Hong JC Liu X Cao W Tai KH Zhao L. Effects of self-efficacy and online learning mind states on learning ineffectiveness during the COVID-19 lockdown. Educ Technol Soc. (2022) 25:142–54. 10.19173/irrodl.v23i2.5775

[55] Jiao W Liu MT Schulz PJ Chang A. Impacts of self-efficacy on food and dietary choices during the first COVID-19 lockdown in China. Foods. (2022) 11:2668. 10.3390/foods11172668 36076852 PMC9455677

